# Metabolomics Provides Novel Insights into the Potential Toxicity Associated with Heated Tobacco Products, Electronic Cigarettes, and Tobacco Cigarettes on Human Bronchial Epithelial BEAS-2B Cells

**DOI:** 10.3390/toxics12020128

**Published:** 2024-02-04

**Authors:** Marie Lenski, Gianni Zarcone, Saïd Maallem, Guillaume Garçon, Jean-Marc Lo-Guidice, Delphine Allorge, Sébastien Anthérieu

**Affiliations:** 1 Univ. Lille, CHU Lille, Institut Pasteur de Lille, ULR 4483, IMPECS—IMPact de l’Environnement Chimique sur la Santé Humaine, F-59000 Lille, France; marie.lenski@univ-lille.fr (M.L.); gianni.zarcone@univ-lille.fr (G.Z.); maallem_said@yahoo.fr (S.M.); guillaume.garcon@univ-lille.fr (G.G.); jean-marc.lo-guidice@univ-lille.fr (J.-M.L.-G.); delphine.allorge@univ-lille.fr (D.A.); 2 CHU Lille, Unité Fonctionnelle de Toxicologie, F-59037 Lille, France

**Keywords:** heat-not-burn products, e-cigarette, lung, metabolites, mass spectrometry

## Abstract

Smoking is an established risk factor for various pathologies including lung cancer. Electronic cigarettes (e-cigs) and heated tobacco products (HTPs) have appeared on the market in recent years, but their safety or, conversely, their toxicity has not yet been demonstrated. This study aimed to compare the metabolome of human lung epithelial cells exposed to emissions of e-cigs, HTPs, or 3R4F cigarettes in order to highlight potential early markers of toxicity. BEAS-2B cells were cultured at the air–liquid interface and exposed to short-term emissions from e-cigs set up at low or medium power, HTPs, or 3R4F cigarettes. Untargeted metabolomic analyses were performed using liquid chromatography coupled with mass spectrometry. Compared to unexposed cells, both 3R4F cigarette and HTP emissions affected the profiles of exogenous compounds, one of which is carcinogenic, as well as those of endogenous metabolites from various pathways including oxidative stress, energy metabolism, and lipid metabolism. However, these effects were observed at lower doses for cigarettes (2 and 4 puffs) than for HTPs (60 and 120 puffs). No difference was observed after e-cig exposure, regardless of the power conditions. These results suggest a lower acute toxicity of e-cig emissions compared to cigarettes and HTPs in BEAS-2B cells. The pathways deregulated by HTP emissions are also described to be altered in respiratory diseases, emphasizing that the toxicity of HTPs should not be underestimated.

## 1. Introduction

The Global Burden of Disease Project [1] estimated that approximately 1.14 billion people worldwide smoked in 2019. The majority of smokers are addicted to nicotine delivered via cigarettes, rationalizing the high prevalence of tobacco-induced diseases decades later [2]. Smoking accounted for 8 million deaths globally in 2023 according to the WHO [3]. The major causes of tobacco death include lung cancer, emphysema, heart attack, stroke, cancer of the upper aerodigestive areas, and bladder cancer [4]. Smoking tobacco is also responsible of chronic diseases such as eye diseases, periodontal diseases, cardiovascular diseases, chronic obstructive pulmonary diseases, diabetes mellitus, rheumatoid arthritis, and disorders affecting the immune system. People who smoke can expect to lose an average of at least a decade of life compared to equivalent non-smokers [5,6]. Smoking cessation greatly reduces the risks of smoking-related diseases. Nicotine administered alone in various nicotine replacement formulations (such as patches or gums) is safe and effective as an evidence-based smoking cessation aid. Novel forms of nicotine delivery systems have also emerged, including electronic cigarettes (e-cigs) and, more recently, heated tobacco products (HTPs). These products are marketed as lower-risk products than conventional cigarettes due to the absence of tobacco for e-cigs or tobacco combustion for HTPs. However, the safety of e-cigs or HTPs has not yet been fully established due to a lack of exhaustive independent toxicological studies.

Our previous findings demonstrated that HTP emissions contained fewer toxic compounds (polycyclic aromatic hydrocarbons, carbonyl compounds, and metals) than conventional cigarette smoke, but more than e-cig aerosols [7,8]. In a model of human bronchial epithelial cells (BEAS-2B cell line) cultured at the air–liquid interface (ALI), conventional cigarettes were more cytotoxic and induced more oxidative stress and genotoxicity than HTPs, unlike e-cigs, which had no effect on these parameters under the studied conditions [9]. In addition to these conventional methods for studying cellular damage, the use of global approaches such as “omics” (metabolomics, transcriptomics, MiRnomics, etc.) can provide a better understanding of the molecular and cellular mechanisms of toxicity, and it helps to identify relevant markers of effects and/or exposure.

Metabolomics aims to comprehensively assess changes in the metabolome induced by endogenous and/or exogenous factors, to screen for significantly different metabolite profiles, and thus to identify potential biomarkers [10]. To date, the metabolic effects of exposure to HTPs or e-cig aerosols have been poorly characterized. A review of the corresponding studies, the experimental conditions implemented, and the main results obtained is summarized in Table 1. Several studies on e-cigs (most of them independent from the tobacco industry) have been carried out in humans [11,12,13,14,15] or using *in vivo* [16,17,18] or in vitro [19,20,21] models. These studies demonstrated the deregulation of many metabolic pathways after e-cig exposure, including glycolysis, the tricarboxylic acid (TCA) cycle, amino-acid metabolism, beta-oxidation, phospholipid metabolism, sphingolipid metabolism, or antioxidant metabolism. Two studies on HTPs showed the benefits of tobacco cessation or switching to HTPs on the human lipidomic lung profile [22,23]. In addition, exposure to HTPs had a lower impact than cigarette smoke on the pathophysiology of human gingival organotypic cultures [24]. However, these few studies were all conducted by the tobacco industry itself. Moreover, it is noteworthy that there are currently no metabolomic studies comparing the toxicity of e-cigs and HTPs in a similar model, although such a comparison would be essential given that both products are increasingly recommended as smoking cessation aids. Therefore, the aim of the present study was to compare the metabolome of human lung epithelial cells (BEAS-2B cell line) exposed to e-cigs, HTPs, or cigarette emissions in order to highlight potential metabolic fingerprints and to identify early markers of toxicity. We applied liquid chromatography–high resolution mass spectrometry (LC-HRMS)-based untargeted metabolomics to analyze endogenous and exogenous compounds in the lysate of cell cultures after HTP, e-cig, or cigarette aerosol exposure.

## 2. Materials and Methods

### 2.1. Chemicals and Reagents

The BEAS-2B cell line was purchased from the American Type Culture Collection (ATCC^®^ CRL9609™ Manassas, VA, USA). LHC-9 medium, phosphate buffer solution (PBS), and type I collagen solution were purchased from Life Technologies (Courtabœuf, France). CellBIND 75 cm^2^ tissue culture flasks and transwell culture inserts (4.67 cm^2^) with a 0.4 µm pore size were bought from Corning (Amsterdam, The Netherlands) and Sigma Aldrich (Saint-Quentin Fallavier, France), respectively. 

3R4F reference cigarettes were purchased from the University of Kentucky (Lexington, KY, USA). The tested HTP was an IQOS 2.4 model manufactured by Philip Morris International (Neuchâtel, Switzerland), used with IQOS heatsticks (amber box from Philip Morris International). A third-generation “ModBox-TC” model manufactured by NHOSS^®^ brand (Bondues, France) was used with an “Air Tank” clearomiser equipped with a 0.5 Ω kanthal coil and containing a “blond tobacco” flavored e-liquid (NHOSS^®^ brand, Bondues, France) with 16 mg/mL of nicotine. 

Water was obtained from a Milli-Q ultrapure water system (Millipore, Burlington, MA, USA). UPLC–MS-grade acetonitrile and methanol were bought from Waters (Milford, MA, USA), and formic acid was purchased from Honeywell (Charlotte, NC, USA). Reagent-grade ammonium formate, β-hydroxy-ethyltheophyllin, and phenobarbital-D5 were purchased from Sigma-Aldrich (St. Louis, MI, USA). Methyl-clonazepam was bought from Roche (LGC, Molsheim, France).

### 2.2. Cell Culture and Experimental Design for Cell Exposure

Human bronchial epithelial BEAS-2B cells were selected as the in vitro model. Their cell culture protocol has already been described [9]. Briefly, the cells were seeded onto transwell culture inserts. The ALI was established by removing the LHC-9 medium from the apical surface, exposing only the basal surface to the medium. The BEAS-2B cells cultured at the ALI were transferred to an exposure module (Vitrocell 6/4 CF module) and exposed to different doses of emissions from the 3R4F cigarette, HTP, or e-cig set up at 18 W (Mb-18W) or 30 W (Mb-30W) using the Vitrocell^®^ VC1 Smoking machine (Vitrocell, Waldkirch, Germany). The cells were not exposed to equal quantities of nicotine for all the devices but to comparable cytotoxicity conditions (>80% cell viability) for all exposures based on preliminary data: 60 and 120 puffs for Mb-18W, Mb-30W, and HTP and 2 and 4 puffs for the 3R4F cigarette [9]. The Health Canada intense (HCI) puff profile was used to test all the products. For HTP exposure, the HCI regime was modified without blocking the ventilation holes of the IQOS heatsticks to avoid overheating of the device. The control cells consisted of unexposed cells that were maintained within the incubator [25]. After exposure, cells were incubated at 37 °C and 5% CO_2_ for 24 h before further sample preparation. The supernatant media were removed and stored at −80 °C until further measurement of lactate dehydrogenase (LDH). Four independent cell cultures were used to replicate each experimental point.

### 2.3. Cytotoxicity Evaluation

Cytotoxicity assays were performed using the Cytotoxicity Detection KitPLUS LDH (Cytotoxicity Detection Kit PLUS, Roche Diagnostics GmbH, Mannheim, Germany) according to the manufacturer’s instructions. The assay relies on the evaluation of LDH activity that is discharged from the cytosol of damaged cells and measured in the supernatant medium 24 h after exposure. A positive control was included: the maximal LDH activity was measured by lysing the cells using Triton-X100. Cytotoxicity was determined as percentages related to positive control cells arbitrarily set at a value of 100%. 

### 2.4. Sample Preparation

For sample harvesting, the cells were washed twice with PBS. Their metabolisms were subsequently quenched by the addition of 800 µL of ice-cold methanol/water (80:20, *v*/*v*). Here, metabolism quenching was achieved by combining a low temperature which decreases enzymatic activities and prevents metabolite spontaneous degradation and through the addition of an organic solvent which inactivates enzymes and contributes to the disruption of cell membranes, thus enabling metabolite extraction [26]. After 20 min of incubation at −20 °C, the cells were scraped off the culture vessel on ice using rubber-tipped cell scrapers. The lysates were collected and subsequently centrifugated for 5 min at 14,000× *g* and +4 °C to eliminate cell debris. The supernatants were recovered in a vial, and the precipitates were rinsed in another 200 µL of ice-cold 80% methanol, vortexed, centrifugated for 5 min at 14,000× *g* at +4 °C, then the supernatant was also transferred to the vial. Quality control samples (QC samples) were prepared by pooling equal aliquots of all the samples. The supernatants were concentrated to dryness using a speedvac, and the dried samples were stored at −80 °C until further use. Just before their injection into the chromatographic system, the samples were reconstituted using 100 µL of a water/methanol (90:10, *v*/*v*) mixture containing internal standards (methyl-clonazepam at 0.125 mg/L, β-hydroxy-ethyltheophyllin at 1.6 mg/L, and phenobarbital-D5 at 1 mg/L in methanol). The samples were then centrifugated for 15 min at 14,000× *g* and +4 °C. The injected volume was set at 10 μL of supernatant for each sample.

### 2.5. LC–MS Conditions

Metabolomic analyses were performed using a Vion IMS-QToF mass spectrometer (Waters, Manchester, UK) controlled with the UNIFI software (version 1.9.4.053 Waters MS Technologies, Manchester, UK). The method has already been described previously [27]. Briefly, analytes were separated using an Acquity™ UPLC HSS T3 column (1.8 μm, 150 × 2.1 mm; Waters) with an Acquity UPLC I-Class system (Waters). The LC–MS sequences were set up as follows: 1× blank (mobile phase A), 1× TestMix (Waters), 5× diluted QC to conditionate the column, 1× QC and diluted QC, 48× samples (randomized, plus 1 QC and 1 diluted QC every 6 samples), 1× QC and diluted QC, 1× TestMix (Waters), 1× blank. Ionization was performed using an electrospray ionization source operating in positive (ESI+) and negative (ESI−) modes. The detection method consisted of untargeted data acquisition (MSe full scan).

### 2.6. Extraction of Raw Data and Pre-Processing

The LC–MS data were analyzed using the Progenesis QI software v1.0.5162 (Nonlinear Dynamics, Newcastle upon Tyne, UK). The retention time alignment, peak picking, and adduct deconvolution were sequentially performed. The sensitivity of the peak-picking step was tuned to recover approximately 5000 features from each analysis. The intensity data for each detected feature were exported from Progenesis QI as CSV files for further data analysis.

### 2.7. Data Processing and Statistical Analysis

Data processing and statistical analyses were conducted in the R environment [28]. When analyzing untargeted metabolomic data, missing values are often encountered, but most multivariate statistical methods cannot be applied when the data have missing values. To use these incomplete data sets, features with more than 50% missing values were removed, and the remaining missing values were replaced by 1/5 of the minimum positive value of each variable (LoDs). The data were then filtered, removing features with relative standard deviations higher than 20% in the QC samples. The data were transformed via log transformation and normalized via cyclic loess normalization to obtain a normally distributed population. The quality of the pre-treatment was assessed and confirmed via principal component analysis and intra-class correlation. Further statistical analyses were performed on the processed data with a significance threshold set at a *p*-value of 0.05 after adjustment for the false discovery rate (FDR) [29] to correct for multiple statistical testing. First, we performed a multivariate supervised partial least squares discriminant analysis (PLS-DA) to discriminate between the different exposures to the 3R4F cigarette, HTP, Mb-18W, and Mb-30W. The features with the highest variable importance in the projection (VIP > 1.5) were selected for a cluster analysis via a multivariate unsupervised analysis heatmap to reveal the relationships of features. Second, an ANOVA test was used to compare the variances of the samples belonging to the 4 different exposure groups. This analysis revealed the deregulated features within each type of exposure. For significant features with a fold-change (FC) > 1.5, a Student’s t-test was performed between the control and exposed samples. The intensity of deregulation compared to the control samples (D0) was expressed as Log2(FC), i.e., Log2(FC_D1_) for deregulation between the control and exposure at the lowest dose (D1) and Log2(FC_D2_) for deregulation between the control and exposure at the highest dose (D2). Significantly deregulated features were considered to be a metabolomic signature resulting from a specific exposure and were submitted to an identification process.

### 2.8. Feature Annotation and Pathway Analysis

Feature identification was performed using the Progenesis QI software. The altered features were further queried to propose feature annotations against three databases, which were, according to their rank of annotating confidence, as follows: (1) a homemade database containing the spectral properties, retention time (Rt), and collision cross section (CCS) for peak annotation from authentic standard compounds [27]; (2) an in-house predicted database containing Rt and CCS predicted using machine learning for almost 114,000 metabolites, as previously described [27]; and (3) a commercial metabolomic profiling CCS library (Waters, Manchester, UK). In a few cases, the spectra were manually checked against the MassBank spectral database [30] to broaden the identification possibilities.

A pathway analysis was carried out using MetaboAnalyst 5.0. The Homo Sapiens (KEGG) pathway library was queried using a hypergeometric test for metabolite enrichment analysis and a relative betweenness centrality topology analysis. The corrected *p*-values (FDR) < 0.25 represented notable enrichments of certain metabolites in a pathway.

## 3. Results

### 3.1. Evaluation of Cytotoxicity

**Figure 1:**
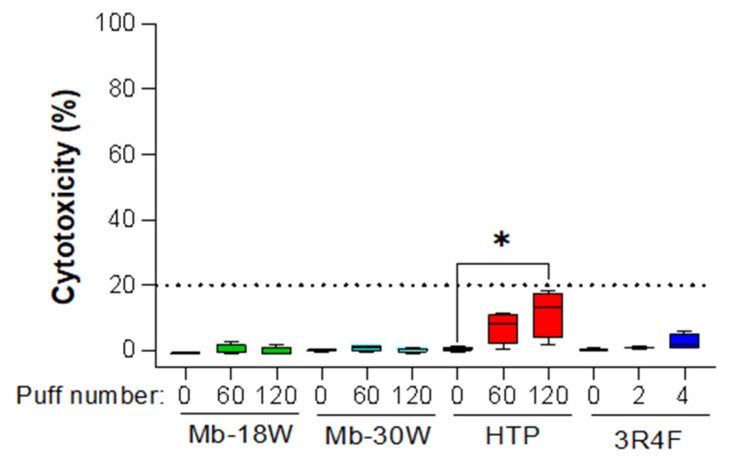
In vitro cytotoxicity of HTP, e-cig (Mb-18W and Mb-30W), and 3R4F cigarette emissions on BEAS-2B cells. Cell viability was evaluated by measuring the LDH released 24 h after exposure. The results are expressed as percentages relative to the LDH released in the cells treated with the positive control (Triton-X100), arbitrarily set at a value of 100%. Types of exposure are: e-cigs [Mb-18W] (green), e-cigs [Mb-30W] (cyan), 3R4F cigarettes (blue) and HTPs (red). The data represent the median and interquartile range from four independent culture replicates. * *p* < 0.05 compared with control cells (Wilcoxon test).

Two exposure doses (D1 and D2) were chosen for each device based on comparable subtoxic doses (>80% cell viability, measured via an ATP test), as previously reported [9], in order to evaluate a potential dose-dependent effect: D1 = 60 puffs and D2 = 120 puffs for Mb-18W, Mb-30W, and HTP and D1 = 2 puffs and D2 = 4 puffs for 3R4F cigarettes. To ensure that the cells were exposed to comparable subtoxic doses in the present metabolomic study, an LDH assay was used to evaluate the cytotoxicity in the cells exposed to the different emissions (Figure 1). HTP emissions caused a higher cytotoxicity after 120 puffs compared to the controls (Kruskal–Wallis test: *p*-value (HTP) = 0.03; Wilcoxon test: *p*-value (D0 vs. D2) = 0.02), while no differences were observed in the other groups. Consistently, we observed that all the devices showed a cytotoxicity below 20%. Thus, under the subtoxic conditions selected for the present metabolomic analysis, the effects of exposure reflected intracellular effects rather than changes due to cell death.

### 3.2. Evaluation of Metabolomics Data Pre-Treatment

Extraction of raw data permitted us to generate a data matrix made up of 5130 and 5037 features in the ESI+ and ESI− modes, respectively. After pre-treatment, the final data matrix was made up of 46 samples (two outlier samples were removed, possibly due to an injection failure) and 3591 features (2398 compounds from ESI+ and 1193 compounds from ESI−). 

### 3.3. Impact of the Type of Emission

**Figure 2:**
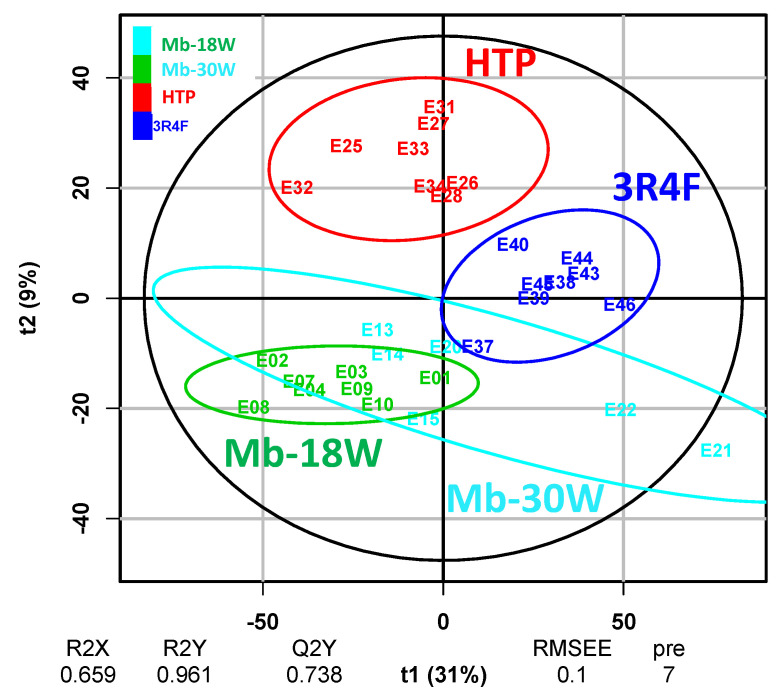
Partial least squares discriminant analysis (PLS-DA). Sample clusters are defined by the type of exposure: 3R4F cigarette (blue), HTP (red), e-cig (green [Mb-18W] and cyan [Mb-30W]). The PLS-DA allowed us to separate the tobacco and the non-tobacco products. Model parameters: components: 7, R2X = 0.659, Q2Y = 0.738, R2Y = 0.961. Validation of the model via permutation test (k = 1000): pQ2 = 0.001, pR2 = 0.001.

A multivariate supervised PLS-DA was first performed to discriminate between the different emissions (3R4F cigarette, HTP, Mb-18W, and Mb-30W). A PLS-DA model was created using the processed data to evaluate group separations and to calculate the VIP scores for each feature. The PLS-DA performed on the exposed groups (D1 and D2 combined) showed strong model statistics for the differentiation of the groups (R2X = 0.659, R2Y = 0.961), with a good reproducibility (Q2Y = 0.738). The obtained loading scatter plot (Figure 2) facilitated a global view of the relationships between variables. This model allowed us to separate the 3R4F cigarette, HTP, and non-tobacco groups (Mb-18W and Mb-30W). 

**Figure 3:**
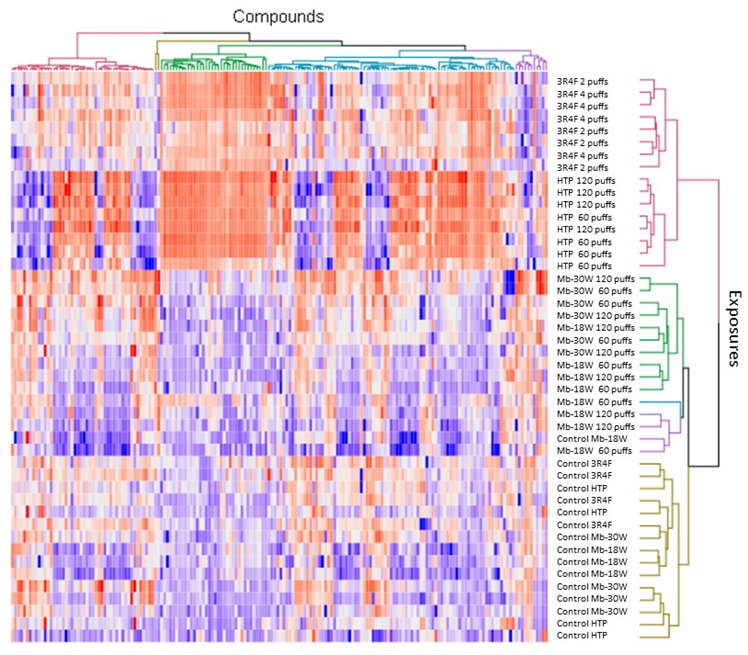
Heatmap analysis showing normalized abundance of discriminant features (VIP > 1.5) during 3R4F cigarette, HTP, Mb-30W, or Mb-18W exposure and in controls (unexposed cells). Red-colored tiles indicate a high abundance and blue indicates a low abundance of the compounds.

The VIP lists were established, pinpointing 180 features with a VIP > 1.5, which were considered to be the discriminant features in this model. These discriminant features were used to build a heatmap (Figure 3). The heatmap analysis revealed a classification of the samples according to two arms. The first arm comprised samples exposed to tobacco products. Among them, those exposed to HTP were separated from those exposed to 3R4F. The second arm consisted of samples exposed to e-cigs or not exposed (controls). Among them, those exposed to e-cigs were fairly well-discriminated from those not exposed. However, the samples exposed to Mb-18W or Mb-30W were not separated. Overall, these data show that both tobacco products induced similar metabolic deregulations, while the metabolome of cells exposed to e-cigs was not very different from that of unexposed cells.

### 3.4. Impact of the Exposure Dose

**Figure 4:**
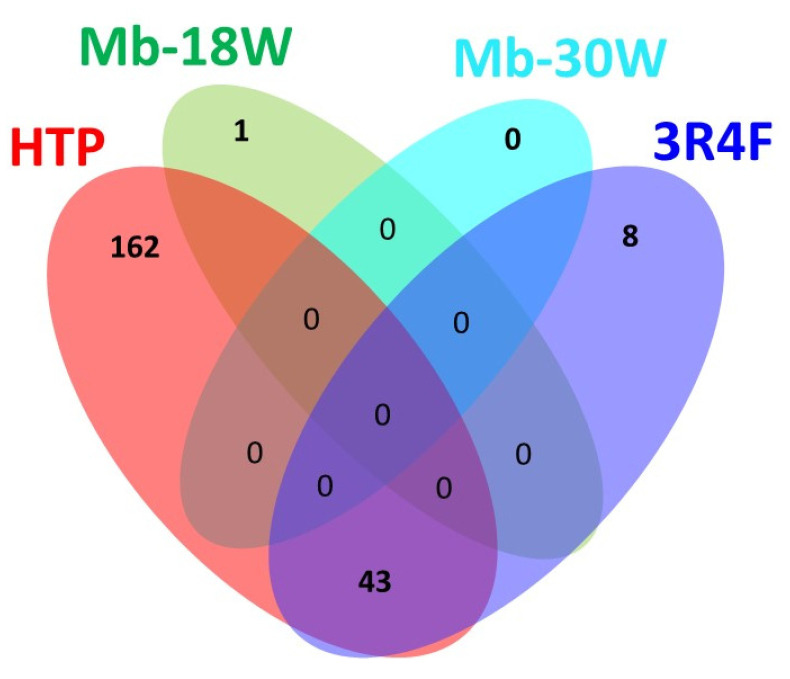
Venn diagram showing the number of significantly selected features based on the ANOVA (FC > 1.5, *p*-value corrected < 0.05) within and common to the 4 exposure groups. Types of exposure are: e-cigs [Mb-18W] (green), e-cigs [Mb-30W] (cyan), 3R4F cigarettes (blue) and HTPs (red).

An ANOVA statistical study was conducted on the pre-treated data with a 95% confidence level (corrected *p*-value < 0.05), demonstrating the presence of significant differences between 214 feature levels in the cells exposed to 3R4F cigarettes, e-cigs, or HTPs compared to unexposed cells. Among them, 95 features were classified at the top of the highest VIP score (VIP > 1.5). A Venn diagram helps to visually represent the number of deregulated features among the four groups (Figure 4). 

Both 3R4F and HTP emissions significantly affected the metabolome of the BEAS-2B cells, whereas no difference was observed after e-cig exposure (only one deregulated compound with Mb-18W) compared to the controls. Notably, 84% of the compounds deregulated after 3R4F exposure were also deregulated after HTP exposure. This corresponded to 43 common compounds. To further identify the impact of the exposure dose, a Student’s t-test was performed (Table 2). A total of 198 and 204 features were deregulated after 60 puffs and 120 puffs of HTP exposure, respectively, out of which 197 were common. Fifty-four percent of the deregulated features were upregulated. Exposure to 2 and 4 puffs of 3R4F cigarette smoke induced a fluctuation of 46 and 51 features, respectively (46 in common). Fifty-one percent of the deregulated metabolites were upregulated. The unique deregulated feature after Mb-18W exposure was upregulated after both durations of exposure.

### 3.5. Feature Identification

The 214 altered features were further queried for compound annotations against databases. The annotation process followed the standards defined by the Metabolomics Standards Initiative [31]. The results are detailed in Table 3. Eleven compounds were identified vs. authentic standards (confidence level = 1), 61 were putatively annotated (confidence level = 2), and 12 were attributed to chemical classes (confidence level = 3). The 84 annotated compounds varied in structure and covered a wide field of endogenous and exogenous metabolites.

### 3.6. Exogenous Compounds

Seven and eight compounds were identified as exogenous compounds after 3R4F and HTP exposure, respectively. Nicotine was significantly increased after exposure to HTP (Log2(FC_D1_) = 16.6; Log2(FC_D2_) = 17.3) or 3R4F (Log2(FC_D1_) = 12.0; Log2(FC_D2_) = 14.3), but not after Mb-18W (*p* = 0.76, Log2(FC_D1_) = 7.6; Log2(FC_D2_) = 8.9) or Mb-30W (*p =* 0.08, Log2(FC_D1_) = 11.9; Log2(FC_D2_) = 12.8). The levels of most of the other exogenous compounds increased compared with the controls, and even more when the exposure dose was higher. This was the case for 1-naphthylamine after exposure to HTP (Log2(FC_D1_) = 15.4; Log2(FC_D2_) = 15.6) or the 3R4F cigarette (Log2(FC_D1_) = 10.0; Log2(FC_D2_) = 12.0), 3-methylindole after exposure to HTP (Log2(FC_D1_) = 4.8; Log2(FC_D2_) = 6.6), and scopoletine after exposure to HTP (Log2(FC_D1_) = 13.1; Log2(FC_D2_) = 13.5) or the 3R4F cigarette (Log2(FC_D1_) = 7.0; Log2(FC_D2_) = 9.5). Regarding the β-carboline family, β-carboline was increased after exposure to HTP aerosols (Log2(FC_D1_) = 9.5; Log2(FC_D2_) = 10.3) or 3R4F cigarettes (Log2(FC_D1_) = 7.6; Log2(FC_D2_) = 10.3), as was harman after exposure to HTP aerosols (Log2(FC_D1_) = 6.7; Log2(FC_D2_) = 7.0) or 3R4F cigarettes (FC_D1_ = 6.4; Log2(FC_D2_) = 7.5). A compound from the pyrroline class, rollipyrrole, was also increased after exposure to HTP aerosols (Log2(FC_D1_) = 11.8; Log2(FC_D2_) = 10.3) or 3R4F cigarettes (Log2(FC_D1_) = 10.9; Log2(FC_D2_) = 11.5). Only one exogenous compound (theaspirane) decreased compared to controls after exposure to either HTP (Log2(FC_D1_) = −2.4; Log2(FC_D2_) = −3.3) or 3R4F cigarettes (Log2(FC_D1_) = −2.0; Log2(FC_D2_) = −2.6). The other significantly deregulated compounds were attributed to endogenous cellular metabolism.

### 3.7. Endogenous Compounds: Pathway Analysis and Biological Interpretation

Of the 51 discriminant features after 3R4F cigarette exposure, 13 were identified as endogenous compounds, including 8 lipids belonging to the eicosanoid class. This small number of identified endogenous metabolites did not permit us to perform an over-representation analysis. 

**Figure 5:**
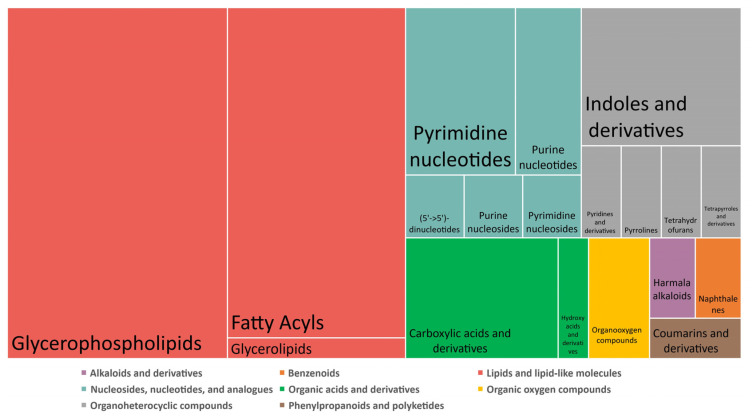
Chemical taxonomy of the identified metabolites deregulated after HTP exposure and the proportion of each super class and class.

Of the 205 discriminant features after HTP exposure, 73 were identified as endogenous compounds including 38 lipids. Their chemical taxonomy and the proportion of each superclass and class are described in Figure 5. Deregulations were mainly linked to metabolism of lipids, carboxylic acids, and nucleotides (purines and pyrimidines). The top 10 metabolic pathways identified after a pathway analysis performed using MetaboAnalyst 5.0 are listed in Table 4. None of the metabolic pathways identified in the KEGG database were significant (*p*-value < 0.05 and FDR < 0.25), possibly due to the small number of identified metabolites, but these results identified metabolic pathways that were potentially deregulated after exposure to HTP.

Several identified metabolites were involved in the amino sugar and nucleotide sugar metabolism. A decrease in uridine diphosphate-*N*-acetylglucosamine (Log2(FC_D1_) = −2.3; Log2(FC_D2_) = −2.0), uridine diphosphate-glucose (Log2(FC_D1_)= −1.4; Log2(FC_D2_) = −1.8) and guanosine diphosphate mannose-mannose (Log2(FC_D1_)= −1.3; Log2(FC_D2_) = −1.5) was observed. Other metabolites were involved in the nucleotide metabolism (purines and pyrimidines). After exposure to HTP aerosols, purine metabolism was mainly affected by an increase in inosine 5′-monophosphate (IMP) (Log2(FC_D1_) = 3.7; Log2(FC_D2_) = 3.7) and a decrease in deoxyguanosine (Log2(FC_D1_) = −1.4; Log2(FC_D2_) = −1.6), and dGTP (Log2(FC_D1_) = −0.7; Log2(FC_D2_) = −1.1). Regarding pyrimidine metabolism, uridine diphosphate (UDP) (Log2(FC_D1_) = −1.4; Log2(FC_D2_) = −1.3), uridine triphosphate (UTP) (Log2(FC_D1_) = −0.8; Log2(FC_D2_) = −1.2), and deoxycytidine (Log2(FC_D1_)= −0.5; Log2(FC_D2_) = −1.0) were down-modulated. Exposure to HTP aerosols also impacted glycerophospholipid metabolism, with an increase in LysoPC(16:0) (Log2(FC_D1_)= 2.5; Log2(FC_D2_) = 3.6) and of CDP-choline (Log2(FC_D1_) = 5.6; Log2(FC_D2_) = 7.0). Finally, we observed an upregulation of an oxidative stress marker, oxidized glutathione (GSSG), after 120 puffs of exposure (Log2(FC_D2_) = 0.9) and a trend towards significance after 60 puffs of exposure (*p*-value = 0.056; Log2(FC_D1_) = 0.48). The increase in oxidized glutathione was associated with a decrease in NADP (Log2(FC_D1_) = −0.4; Log2(FC_D2_) = −0.7), both involved in glutathione metabolism.

## 4. Discussion

Considering the effects of tobacco smoke on health and lifespan and the limited information on the biological/health effects of e-cigs and HTPs use by consumers, there is a need to evaluate the health risks of these new tobacco and vaping products and to identify markers that would help us to understand their underlying physiopathological processes. In this study, we used metabolomic profiling to examine and compare the metabolic responses of a bronchial epithelial cell model to short-term exposure to cigarette smoke (2 or 4 puffs), HTP emissions (60 or 120 puffs), or e-cig aerosols (60 or 120 puffs) generated by a device set up at two powers (18 W or 30 W). The doses of exposure were chosen based on prior studies showing that exposure can cause cell death after higher doses of exposure to HTPs and 3R4F cigarettes [9]. Here, the cytotoxicity assessed by measuring LDH in the culture media of the samples included in this study was consistent with these preliminary data, with a cytotoxicity <20% compared with the control samples. Working at low and comparable cytotoxicity conditions ensured that the differences observed in metabolite abundance reflected biological variability due to exposure and not due to cell death.

We differentially analyzed the 10,167 features detected using LC-HRMS. We were able to filter 214 differences between the exposed and control samples. Based on the metabolites that were significantly deregulated (FC > 1.5 and adjusted *p*-value < 0.05), a robust metabolomic fingerprint composed of 51 or 205 features was shown to be linked to 3R4F or HTP exposure, respectively. The number of significantly deregulated compounds and the intensity of these modulations (FC) increased to a limited extent with the dose of exposure, suggesting that there was a small dose-dependent effect. Eighty-four percent of the discriminant compounds after 3R4F exposure were also discriminant after exposure to HTP aerosols, suggesting common markers of exposure or effects between both tobacco products. These effects are detectable after lower-exposure doses for cigarettes (<4 puffs) than for HTPs (60 and 120 puffs). The greater number of discriminant metabolites after HTP exposure can be likely explained by the difference in exposure doses between HTP (60 and 120 puffs) and the 3R4F cigarette (2 and 4 puffs). Exposure to HTPs and e-cigs was more comparable, as the same doses were used for both devices. Only 1 metabolite was significantly deregulated after exposure to 60 or 120 puffs from Mb-18W, while no features were significantly deregulated with Mb-30W. This unidentified compound was not significantly modulated in the other exposure conditions. This suggests that there were few measurable metabolic alterations in our experimental conditions after exposure to e-cigs. 

Among the 214 differential features, we identified 84 exogenous or endogenous compounds: 11 identified compounds (level 1), 61 putatively annotated compounds (level 2), and 12 putatively characterized compound classes (level 3). One hundred and thirty features (60% of the deregulated features) remained as unknown signals (level 4), which is not surprising given that feature identification is one of the main limitations of untargeted metabolomics. These annotations allowed for powerful deciphering of deregulated compounds to better understand the cellular effects of tobacco or HTP exposure. 

Of the compounds identified, eight were exogenous compounds, seven of which varied in a similar way when exposed to 3R4F smoke or HTP emissions. Another compound, 3-methylindole, was only increased through exposure to HTP emissions. In addition to nicotine, we highlighted an increase in the exogenous compounds that were already described in the literature as originating from the tobacco plant itself or from tobacco smoke: 1-naphthylamine (aromatic amines, naphthalene class) [32,33], scopoletin (polyphenol, coumarins, and derivatives class) [34], norharman, and harman (β-carbolines) [35]. The compound 3-methylindole, which was increased after exposure to HTP aerosols but not after exposure to 3R4F smoke (potentially due to the low exposure dose for 3R4F), belongs to the indole and derivatives class. It is formed through the pyrolysis of tryptophan during tobacco combustion. This result could be in favor of a pyrolysis phenomenon occurring in response to the HTP device. The results published by Vivarell *et al*. indicate that HTP emissions contain pyrolysis and thermal degradation by-products identical to conventional cigarette smoke, and that they cause serious lung damage and increase the risk of cancer in animal models [36]. The compound 3-methylindole has been described as cytotoxic in BEAS-2B cell lines after bio-activation [37] and as mutagenic in human lung microsome models, supporting the hypothesis of a probable pulmonary carcinogenic effect in humans [38]. In normal human bronchial epithelial (NHBE) cells, exposure to 3-methylindole also caused significant DNA damage and mutations without triggering apoptotic defenses, reinforcing the hypothesis that this compound inhaled from cigarette smoke could be a selective lung carcinogen [39]. In conclusion, these exogenous compounds can be considered as markers of exposure to tobacco products, both from cigarette and HTP emissions. While the tobacco industry described HTP as riskless to users’ health, our results suggest that they could be nonetheless toxic, as one carcinogenic compound was identified. The toxicity of HTP should therefore not be underestimated. 

Exposure to tobacco products was associated with a pulmonary cellular stress response, notably an oxidative stress response directly caused by exposure to chemical compounds or induced by the generation of reactive oxygen species (ROS). First, we observed disturbances in glutathione metabolism, notably with an increase in oxidized glutathione after exposure to the higher dose, both for HTP and 3R4F emissions. These results support the generation of oxidative stress following exposure to tobacco products. These findings were consistent with our previously published results, which showed an increase in the oxidized to reduced glutathione ratio following exposure to cigarette and HTP emissions [7]. We also showed that HTPs and 3R4F (but not e-cigs) induced activation of the transcription factor Nrf2 and expression of its target genes, heme oxygenase 1 and NAD(P)H-quinone dehydrogenase 1, demonstrating an antioxidant response after exposure to tobacco products [9]. In our metabolomic study, an increase in biliverdin was observed after exposure to 3R4F emissions (two and four puffs). Biliverdin is a heme metabolite produced under the action of a cryoprotective enzyme, heme oxygenase (HO) [40,41]. Biliverdin is then rapidly converted to bilirubin by biliverdin reductase. Biliverdin can be regenerated from bilirubin through reactions with ROS. Various properties have been attributed to biliverdin and bilirubin, including antioxidant properties in response to oxidative stress. A study by Titz et al. [23] analyzing the metabolome of lung tissue after exposure of mice to conventional cigarettes or HTPs also revealed deregulation of bilirubin metabolism after exposure to cigarettes but not to HTP. Second, our study also revealed modulations in nucleotide metabolism (purines and pyrimidines) after exposure to HTPs (but not after 3R4F cigarettes). Purine metabolism has been reported to produce ROS via the xanthine oxidase pathway [42]. In addition, a deregulation in nucleotide metabolism has already been described in cigarette smokers, with a role in cancer development [43,44]. Third, we observed a decrease in methionine after exposure to HTP. Oxidative stress can induce methionine oxidation [45], leading to a decrease in methionine. An increase in the products of this oxidation (methionine sulphoxide and *N*-acetyl-methionine sulphoxide) has been described following exposure of human gingival epithelial cells for 28 min over 3 days to conventional cigarette smoke, but not to HTP emissions [24]. A decrease in plasmatic methionine has also been observed in cigarette users compared with people who vaper or unexposed individuals [13].

The deregulation of several metabolites indicated an alteration in energy metabolism following exposure to tobacco products. Increased energy demand is an expected response for all cells under stress. Thus, ADP levels were increased after exposure to HTP aerosols. An intermediate of the TCA cycle, isocitrate, was decreased in cells exposed to HTP compared with controls. It is well known that cigarette smoke inhibits mitochondrial respiratory function and deregulates the TCA cycle [46], a central pathway for cellular energy metabolism. Isocitrate had also been previously described as decreased after repeated exposure of 3D bronchial tissue culture to cigarette smoke [47]. Our analysis also showed a significant decrease in isovalerylcarnitine, a short-chain carnitine, and a decrease in an intermediate-chain acylcarnitine, hexanoylcarnitine, after exposure to HTP emissions. Acylcarnitines are organic compounds containing a fatty acid. They play a central role in the transport of fatty acids across the inner mitochondrial membrane during beta-oxidation, the metabolic pathway through which fatty acids are broken down to produce acetyl-CoA, which feeds the TCA cycle. Their disruption confirms mitochondrial dysfunction and disruption of beta-oxidation of fatty acids after exposure to tobacco products, which may reflect a high intracellular energy demand.

**Figure 6:**
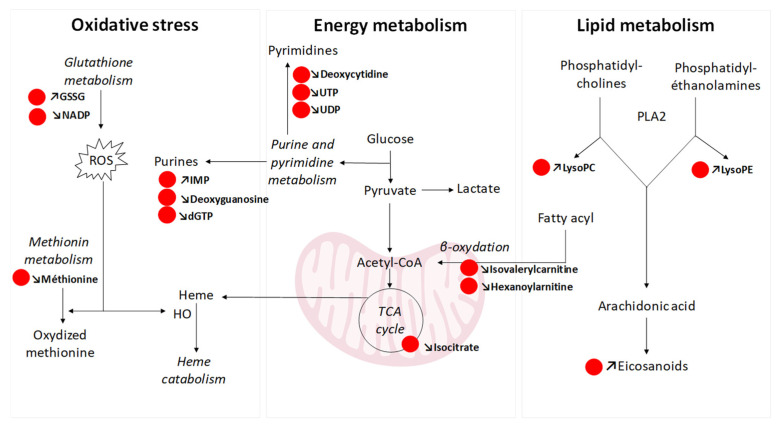
Schematic overview of the distributed metabolic pathways in BEAS-2B cells upon HTP exposure. Metabolites marked in red represent the metabolites affected by HTP exposure. dGTP: deoxyguanosine triphosphate; GSSG: oxidized glutathione; HO: heme oxygenase, IMP: inosine 5′-monophosphate; LysoPC: lysophosphatidylcholine; LysoPE: lysophosphatidylethanolamine; NADP: nicotinamide adenine dinucleotide phosphate; PLA2: phospholipase A2, ROS: reactive oxygen species; UDP: uridine diphosphate; UTP: uridine triphosphate.

Lipids other than carnitines were also affected by exposure to tobacco products (Figure 6), with significant variations in several classes of lipids such as glycerophospholipids (glycerophosphocholines (LysoPC) or glyceroethanolamines (LysoPE)) or fatty acids (carnitines, eicosanoids). Most of these lipids increased in a dose-dependent manner in favor of lipid accumulation. Eicosanoids, derived from arachidonic acid through the action of the lipooxygenase and cyclooxygenase enzymes, are signaling mediators of the inflammatory response. An increase in the activity of these enzymes had already been described after HTP exposure [36], but also in smokers [48], and was associated with inflammation and pulmonary pathologies [49] and cancers [50]. Other lipid classes were also increased after exposure to HTP emissions. These included lysoglycerophospholipids of the glycerophosphocholine subclass (lysophosphatidylcholines, LysoPC) and glycerophosphoethanolamines (lysophosphatidylethanolamines, LysoPE). LysoPCs are produced through the cleavage of phosphatidylcholine by phospholipase A2 (PLA2), a reaction that also forms free fatty acids such as arachidonic acid, which is the precursor of eicosanoids [51]. This reaction is also possible under the effects of ROS. LysoPEs are also produced through the cleavage of phosphatidylethanolamine by PLA2. Altogether, exposure to cigarette and HTP emissions could therefore affect the regulation of the inflammatory response in bronchial cells. Studies conducted by the tobacco industry comparing the murine bronchial tissue lipidome after exposure to tobacco and HTP have shown lipid deregulation, but much more was marked for cigarettes than for HTP (very limited effects for HTP), allowing them to conclude that HTP had a lower toxic effect than conventional cigarettes [22,23]. Given our results, the impact of HTP on those metabolic pathways should not be underestimated.

Some limitations of the present study must be mentioned. A unique regime (HCI) was used for all the tested products to compare their toxicity under the same laboratory conditions. However, other standardized smoking or vaping regimes have been created based on users’ puffing behavior (ISO 20768:2018 [52] for e-cigs, HCI and ISO3308:2012 [53] for tobacco products). In this study, the unique regime choice could be a limitation to an accurate assessment of potential health implications in the context of different product categories. In addition, the nontargeted metabolomic analysis is an initial screening. Level 2 annotations should be interpreted with caution, as some of them may derive from a false positive annotation. In this context, further target verification is essential in the future. In particular, it should be noted that the majority of studies analyzing eicosanoids use analytical techniques with a negative ionization mode, whereas here, the identifications were made for compounds detected using a positive ionization mode. In this context, and given the risk of erroneous identifications, the confirmation of these identifications will be essential to confirm these conclusions. By using our in vitro model, we were able to evaluate the acute effects of exposure exclusively. While no significant deregulation of metabolites was found after e-cig acute exposure in the present study, other researchers have demonstrated modifications in the metabolomes in the urine, plasma, or saliva of people who vape [11,13,14,15], or in mice chronically exposed to e-cigs [16,17,18]. Therefore, this comparative study of the acute toxicity of e-cigs and HTPs using a metabolomic approach will need to be completed by animal or population studies in order to compare the long-term toxicity of these new tobacco and vaping devices.

## 5. Conclusions

This study provides innovative data to compare the acute toxicity of alternative tobacco products (e-cigs and HTPs) and to better understand their cellular and molecular mechanisms of toxicity. The metabolomic data observed strongly suggest a lower acute toxicity of e-cig aerosols compared to cigarette and HTP emissions in the BEAS-2B cell line. The metabolomic fingerprint identified for both tobacco products (HTP and 3R4F) consisted of exogenous compounds, one of which is carcinogenic, as well as endogenous metabolites, which can be considered as markers of effects. Their deregulations, which are only observed after more intensive exposure to HTPs (60 or 120 puffs) than to cigarettes (2 or 4 puffs), indicate alterations in various metabolic pathways, including oxidative stress and mitochondrial and lipid metabolisms. The metabolites deregulated by HTPs are involved in metabolic pathways that are also altered in respiratory diseases, confirming that the toxicity of HTPs should not be underestimated. Further long-term studies in animal models should be conducted to allow for the assessment of chronic exposures to HTPs. This work provides health agencies and authorities with additional information for the regulation of these products as well as for the development of public health policies to reduce smoking and tobacco product consumption.

## Figures and Tables

**Table 1:** Literature review summary of the effects of e-cig or HTP aerosols on host metabolism.

Biological Question	Exposure Device Duration	Model Sample Type Sample Size	Deregulated Metabolite(s)/Metabolic Pathway(s) *	Conclusions	Independent Study	Ref
Cessation of vaping in regular heavy e-cigarette users	4th e-cig generation Short-term (5 days)	Human Urine, plasma *n* = 30	Serum: no difference. Urine: A specific metabolomic signature characterized the stop-session, including 3-hydroxyisovalerate (↘), pyruvate (↘), trimethylamine oxide (↗), hippurate (↗), and N-phenylacetyl-glycine (↗).	In regular e-cig users, short-term vaping cessation shifted baseline urine metabolome	Yes	[11]
Switch from cigarette to e-cig	1st e-cig generation Short-term (5 days)	Human Urine, plasma *n* = 75	↘ xenobiotic exposure (nicotine and its metabolites, other cigarette smoke constituents). Improved vitamin metabolism and ↘ oxidative stress.	Less toxic environment for consumers of e-cigs and potential health benefits compared to people who smoke cigarettes	No	[12]
Effects of chronic e-cig vaping and cigarette smoking	No information on e-cig exposure device Long-term (>2 years)	Human Plasma *n* = 24	E-cig vaping deregulated TCA cycle-related metabolites, while cigarette smoking altered sphingolipid metabolism.	Specific metabolic signatures could serve as potential systemic biomarkers for early pathogenesis of cardiopulmonary diseases	Yes	[13]
Long-term effects of e-cigs compared to tobacco	No information on e-cig exposure device Long-term effects (>6 months)	Human Urine *n* = 117	Metabolomic signature of 839 and 396 features for people who smoke and vape, respectively, including 12% of common metabolites. ↗ acylcarnitines and acylglycines in vapers, suggesting higher lipid peroxidation. Trend of ↗ in cancer-related biomarkers (Me-Fapy) in people who vape.	Deregulation of markers of inflammatory status and fatty acid oxidation in people who vape, as well as a trend of elevated cancer-related biomarkers.	Yes	[14]
Effects of e-cigs on oral health	4th e-cig generation Long-term (one month to 2 years)	Human Saliva *n* = 30	Perturbation of 368 metabolites in vapors. ↗ prostaglandins, ↗ leukotrienes (arachidonic acid metabolism). Alterations in immune signaling metabolites (gangliosides, ceramides, angiotensin).	Potential biomarkers of periodontal disease in vapors.	Yes	[15]
Acute exposure to e-cigs	4th e-cig generation Short-term (1 h to 8 h)	Mouse Serum *n* = 40	Deregulation of 26 to 50 metabolites after exposure. The type of compound changed over time. Total of 24 metabolic pathways affected, mainly regulated amino acid metabolism, further affected the TCA cycle.	Highlight specific metabolic signatures of e-cigs acute exposure that are potentially beneficial for disease prevention	Yes	[16]
Long-term effects of e-cigs with or without nicotine	2nd e-cig generation Long-term (4 months)	Mouse Bronchoalveolar lavage *n* = 9	Independently of nicotine presence, altered lung lipid homeostasis in alveolar macrophages and epithelial cells. Aberrant phospholipids in alveolar macrophages and increased surfactant-associated phospholipids in the airway. Downregulation of innate immunity against viral pathogens in resident macrophages.	Alterations in lipid homeostasis and immune impairment are independent of nicotine, thereby warranting more extensive investigations on the vehicle solvents used in e-cigs	Yes	[17]
Effects of the type of e-cig consumption	2nd, 3rd, 4th e-cig generation 4 to 12 weeks	Mouse Plasma *n* = 6	Different alterations in metabolomic profiles depending on the e-cig generation, chemical compounds, duration of exposure, and gender. These signatures have been associated with cardiovascular diseases and can serve as predictors of chronic kidney diseases.	Each e-cig generation and each e-liquid are likely to lead to their own set of health effects.	Yes	[18]
Effects of e-liquid compared to cigarette smoke condensate	E-liquid or cigarette smoke condensate Short-term (1 h to 13 h)	HBEC * (at ALI) Intracellular content *n* = 3	E-liquid and cigarette smoke condensate affected 24% and 35% of the metabolome, respectively, with biphasic fluctuations: first maximum after 5 h, second maximum after 13 h. Alterations in amino acids, energy, β-oxidation of fatty acid metabolism.	E-liquid profoundly alters the metabolome of HBEC in a manner which is comparable and partially overlapping with the effects of cigarette smoke condensate	Yes	[19]
Effects of e-cig vanillin (flavorant)	e-liquid on cells Short-term (18 h)	BEAS-2B cell line Intracellular content *n* = 3	Vanillin perturbed specific energy, amino acid, antioxidant, and sphingolipid pathways previously associated with human disease such as lung disease including asthma, idiopathic pulmonary fibrosis, and acute respiratory distress syndrome.	Vanillin could drive the lung metabolic microenvironment to a more pathogenic state.	Yes	[20]
Effects of e-cig maltol (flavorant)	3rd e-cig generation Short-term (1 h)	BEAS-2B cell line Intracellular content *n* = 3	Perturbation of oxidative stress with e-liquids with or without maltol. Deregulation of amino acid metabolism specifically with maltol. Many effects of firsthand exposure were also observed with secondhand exposure.	Flavorants in e-liquids impact lung metabolism after both firsthand and secondhand exposure.	Yes	[21]
Switch from cigarettes to HTPs or smoke cessation	HTP Long-term (2–8 months)	Mouse Lung intracellular content *n* = 8	↗ candidate surfactant lipids, ↗ inflammatory eicosanoids, ↗ ceramide classes after cigarette exposure that were absent in mice from the cessation group and the switching group to HTPs.	Benefits of tobacco cessation or switching to an HTP for lipidomic lung profile.	No	[22]
Switch from cigarettes to HTPs or smoke cessation	HTP Long-term (6 months)	Mouse Lung intracellular content *n* = 9	Substantial effects of 3R4F exposure: ↗ inflammatory and oxidative stress responses, ↗ metabolites with immunoregulatory roles (itaconate, polyamines, quinolinate), ↗ metabolites of oxidative stress response (heme–biliverdin–bilirubin pathway). HTP aerosol exposure was associated with fewer to absent effects.	Benefits of tobacco cessation or switching to an HTP for metabolic lung profile	No	[23]
Effects of HTPs compared to tobacco	HTP Short-term (3 days)	Human gingival epithelial cells Intracellular content *n* = 5	13 metabolites perturbed after HTP exposure vs. 181 for cigarettes. Reduction in the metabolic impact in HTP aerosol-exposed samples with respect to cigarettes.	Exposure to HTP aerosol had a lower impact on the pathophysiology of human gingival organotypic cultures than cigarette smoke	No	[24]

* ↘: reduction; ↗: elevation; HBEC: human bronchial epithelial cells.

**Table 2:** Number of significantly deregulated compounds for each type of exposure after t-test analysis (corrected *p* (FDR) < 0.05).

Type of Exposure	D0 *vs.* D1	D0 *vs.* D2	Common Compounds
3R4F	46	51	46
HTP	198	204	197
Mb-18W	1	1	1
Mb-30W	0	0	0

**Table 3:** List of the 84 metabolites identified via metabolomic analysis in the positive (POS) and negative (NEG) ionization mode (ESI) and their associated Log2(FC) between the control (D0) and exposure to the lowest dose (D1) or the highest dose (D2). The values highlighted in grey indicate significant metabolites (Student’s *t*-test; corrected *p* < 0.05).

ESI	Peak	Confidence Level	HMDB	Name	HTP Log2(FC) D0 *vs*.		3R4F Log2(FC) D0 *vs*.		Mb-18W Log2(FC) D0 *vs*.		Mb-30W Log2(FC) D0 *vs*.	
					D1	D2	D1	D2	D1	D2	D1	D2
NEG	0.93_565.0441*m/z*	2	HMDB0000286	Uridine diphosphate glucose	−1.3	−1.7	−0.3	−1.1	−0.1	−0.5	0.1	0
NEG	0.97_607.0776n	2	HMDB0000290	Uridine diphosphate-*N*-acetylglucosamine	−1.1	−1.2	−0.1	−0.7	−0.3	−0.3	−0.7	−0.7
NEG	0.97_628.0517*m/z*	2	HMDB0000290	Uridine diphosphate-*N*-acetylglucosamine	−2.3	−2	−0.2	−1.1	−0.3	−0.3	−1.3	−1.2
NEG	1.00_482.9586*m/z*	1	HMDB00285	Uridine triphosphate	−0.8	−1.2	−0.5	−1.5	0	−0.4	0.3	0
NEG	1.00_506.9926n	2	HMDB0001440	dGTP	−0.5	−1	−0.4	−0.8	−0.2	−0.4	0.3	0
NEG	1.04_191.0547*m/z*	1	HMDB03072	Quinic acid	2.8	3	1.1	2.3	1.2	1	−0.4	0
NEG	1.08_427.0267n	1	HMDB00061	Adenosine 3′,5′-diphosphate	1.1	1.5	0.4	1.1	0.3	0.1	0	0
NEG	1.08_604.0656*m/z*	1	HMDB01163	Guanosine diphosphate mannose	−1.2	−1.4	−0.3	−0.6	−0.5	−0.4	−0.9	−0.5
NEG	1.09_429.0553*m/z*	2	HMDB0060067	CMP-2-aminoethylphosphonate	−0.6	−1.3	−0.3	−0.5	0	0	0.5	0.1
NEG	1.59_742.0631*m/z*	1	HMDB00217	NADP	−0.4	−0.7	−0.1	−0.3	0.1	−0.4	0	−0.3
NEG	1.60_347.0374*m/z*	1	HMDB00175	Inosine 5′-monophosphate	3.6	3.7	1.5	3.6	0.4	0	1.4	1
NEG	1.64_148.0420*m/z*	1	HMDB00696	Methionine	−1.2	−1.2	−0.2	−0.4	−0.5	−0.5	−0.2	−0.8
NEG	2.35_321.0676n	2	HMDB0013220	Beta-citryl-l-glutamic acid	−1	−1.2	−0.5	−0.6	−0.3	−0.5	−0.1	−0.3
NEG	2.96_612.1481n	2	HMDB0003337	Oxidized glutathione	0.3	0.7	0.2	1.2	0.1	0.1	0.1	0.2
NEG	14.11_498.2602*m/z*	2	HMDB0011519	LysoPE 20:5	2.3	2.7	0.8	0.3	0.3	0.6	0.5	0.4
NEG	14.98_526.2911*m/z*	2	HMDB0011525	LysoPE 22:5	2.4	2.9	0.7	0.4	0.2	0.3	0.2	0.1
NEG	15.47_506.3213*m/z*	2	HMDB0011512	LysoPE 20:1	0.9	1.4	0.2	0.5	0.4	0.3	0.1	−0.1
NEG	15.69_506.3213*m/z*	2	HMDB0011512	LysoPE 20:1	0.9	1.8	0	0.3	0.4	0.2	0.3	0.2
POS	0.96_404.0019n	2	HMDB0000295	Uridine 5′-diphosphate	−1.3	−1.2	−0.4	−0.5	−0.3	−0.1	−0.4	−1.1
POS	0.98_489.1138*m/z*	1	HMDB0001413	Cytidine 5′-diphosphocholine	5.5	6.9	0.7	3.9	0.7	1.7	−0.2	1
POS	0.99_506.9954n	2	HMDB0001440	dGTP	−0.6	−1.1	−0.1	−0.4	−0.3	−0.3	0	0
POS	1.07_192.0265n	1	HMDB0000193	Isocitric acid	−1	−1.3	−0.4	−0.3	−0.3	−0.1	0	−0.1
POS	1.07_321.0689n	2	HMDB0013220	Beta-citryl-l-glutamic acid	−1.3	−1.4	−0.6	−0.7	−0.4	−0.2	−0.3	−0.5
POS	1.07_612.1507n	2	HMDB0003337	Oxidized glutathione	0.3	0.5	0	1.4	0	0.2	0.1	0
POS	1.11_250.0931*m/z*	2	HMDB0000085	Deoxyguanosine	−1.4	−1.5	−0.6	−0.8	−0.1	0	0	−0.3
POS	2.01_227.0902n	1	HMDB00014	Deoxycytidine	−0.5	−1	−0.2	−0.2	−0.1	0	0.3	0.1
POS	2.29_321.0690n	2	HMDB0013220	Beta-citryl-l-glutamic acid	−1.3	−1.5	−0.5	−0.7	−0.4	−0.1	0.1	−0.5
POS	2.98_612.1497n	2	HMDB0003337	Oxidized glutathione	0.4	0.9	0.2	1.7	0	0.3	0.4	0.2
POS	3.63_132.0806*m/z*	2	HMDB0000466	3-Methylindole	4.8	6.6	1.9	2.3	4	4.5	4.3	3.3
POS	3.82_229.1781n	2	HMDB0041947	N1,N8-diacetylspermidine	4.6	6.8	2.8	6.5	4.2	4.4	1.5	2.8
POS	3.83_230.1859*m/z*	2	HMDB0041947	N1,N8-diacetylspermidine	3.1	4.9	1.7	4.2	1.5	1.7	1.1	1.9
POS	4.22_221.1280*m/z*	2	HMDB0002096	3-indolebutyric acid	8.6	9.6	6.2	8.7	2.1	0.9	0	0.4
POS	4.53_143.0734n	2	HMDB0243964	1-naphthylamine	15.3	15.6	9.9	11.9	5	7.5	4.5	6.6
POS	5.62_163.1228*m/z*	1	HMDB0001934	Nicotine	16.5	17.2	11.9	14.3	7.6	8.9	11.9	12.8
POS	6.54_191.1176*m/z*	2	HMDB0004369	N-methylserotonin	10.5	11.3	6.5	9	2.3	3.9	−0.3	1.2
POS	6.59_190.0840n	2	HMDB0000325	3-hydroxysuberic acid	6.6	6.8	1.9	3.2	−0.1	−1	1.7	3.2
POS	7.26_187.0629n	2	HMDB0000734	Indoleacrylic acid	−0.9	−0.8	−0.2	−0.2	−0.3	−0.2	0	−0.5
POS	8.18_246.1697*m/z*	2	HMDB0000688	Isovalerylcarnitine	−0.9	−1.4	−0.7	−1.3	−0.5	−0.3	−0.2	−0.6
POS	8.53_169.0760*m/z*	2	HMDB0012897	Beta-carboline	9.5	10.2	7.6	8.8	1.7	2	0.6	0.1
POS	8.82_183.0914*m/z*	2	HMDB0035196	Harman	6.6	7	6.4	7.5	0.1	0.6	−0.6	−0.5
POS	9.00_215.1177*m/z*	2	HMDB0001389	Melatonin	11.1	12.4	5.1	7.5	3.5	4.9	6.9	7.1
POS	9.22_260.1851*m/z*	2	HMDB0000705	Hexanoylcarnitine	−1	−0.9	−0.6	−0.7	−0.1	0	0.5	0.2
POS	9.34_193.0494*m/z*	2	HMDB0034344	Scopoleptin	13.1	13.4	6.9	9.5	−0.6	−1	−0.2	0.1
POS	11.43_236.2004*m/z*	2	HMDB0036823	Theaspirane	−2.4	−3.2	−2	−2.5	−1	−1	−0.8	−1.3
POS	12.54_262.1781n	2	HMDB0032297	Glyceryl 5-hydroxydecanoate	0.6	1.6	−0.1	1	1.9	2.9	0.6	1
POS	12.78_391.1874*m/z*	3	-	Eicosanoid	11.3	11.8	6.6	8.9	−0.5	1	−0.9	−0.6
POS	12.85_253.1335*m/z*	2	HMDB0037554	Rollipyrrole	11.7	10.3	10.9	11.5	−5.9	−5.7	3.7	−0.1
POS	12.93_377.2081*m/z*	3	-	Eicosanoid	11.1	10.9	10	10.8	4.9	5.7	−9.3	−0.7
POS	13.03_391.1889*m/z*	3	-	Eicosanoid	5.3	6.4	2	4.1	0	0.5	0	0
POS	13.15_391.1875*m/z*	3	-	Eicosanoid	9.2	9.6	2.7	5.7	0.9	0.9	2.1	1
POS	13.17_377.2074*m/z*	3	-	Eicosanoid	12.1	11	8.4	8.3	11.4	2.1	2.8	2.2
POS	13.36_336.2287n	3	-	Eicosanoid	9.5	10.4	6.3	8.2	0.4	1.7	0.4	1
POS	13.37_465.2850n	2	HMDB0010380	LysoPC(14:1)	2.1	2.8	0	0.5	0.4	0.8	0.2	0.7
POS	13.52_377.2078*m/z*	3	-	Eicosanoid	11.4	11.4	6.6	9.3	3	2.4	−1.1	0.9
POS	13.61_583.2540*m/z*	2	HMDB01008	Biliverdin	1.7	0.3	1.5	2.1	−0.3	0.4	0	0.1
POS	13.86_467.3004n	2	HMDB0010379	LysoPC(14:0/0:0)	1.3	1.6	0.3	0.4	0.2	0.5	0.3	0.4
POS	13.97_359.1982*m/z*	3	-	Eicosanoid	9.3	9.2	3.2	5	0.1	−0.6	1.7	1.5
POS	14.07_499.2692n	2	HMDB0011489	LysoPE(0:0/20:5)	2.4	3	0.4	0.5	0.3	0.9	0.6	1.3
POS	14.12_541.3164n	2	HMDB0010397	LysoPC(20:5)	5.5	7.1	0.8	1.5	1.1	1.5	2.3	0.9
POS	14.18_373.1770*m/z*	3	-	Eicosanoid	14.9	13.9	4.1	5.3	−5.1	−10.7	0.2	5.9
POS	14.24_493.3160n	2	HMDB0010383	LysoPC(16:1/0:0)	1.2	1.7	0	0.1	0	0.2	0	0.2
POS	14.27_518.3212*m/z*	2	HMDB0010382	LysoPC(16:0)	2.4	3.5	−0.3	0.4	1	1.6	1.4	0.7
POS	14.42_359.1980*m/z*	3	-	Eicosanoid	17.1	16	10.5	11.5	1.5	−4.6	0	0
POS	14.66_322.2497n	3	-	Eicosanoid	9.4	8.8	0	2.3	−1	5.5	2.1	4.3
POS	14.68_519.3316n	2	HMDB0010386	LysoPC(18:2)	2	2.9	0	0.4	0	0.5	0.3	0.5
POS	14.72_543.3316n	2	HMDB0010395	LysoPC(20:4)	2.9	4.2	0.1	0.7	0.4	0.6	1.1	0.4
POS	14.83_508.3388*m/z*	2	HMDB0012108	LPC 17:1	1.9	2.5	−0.3	0.4	0.9	1.6	−0.1	0.5
POS	14.85_510.3983*m/z*	2	HMDB0072866	MG(10:0/0:0/0:0)	−3.2	−7.3	−1.5	−4.4	−0.9	−0.1	1.3	−2.1
POS	14.96_519.3317n	2	HMDB0010386	LysoPC(18:2)	1	1.5	−0.3	−0.1	0.2	0.3	0.2	0.4
POS	15.00_569.3475n	2	HMDB0010403	LysoPC(22:5)	4.6	6.5	0.4	1.3	0.9	1	1.8	1.3
POS	15.07_495.3320n	2	HMDB0010382	LysoPC(16:0)	0.7	1.1	0.1	0.1	0.1	0.3	0.5	0.3
POS	15.21_584.3104*m/z*	2	HMDB0010393	LysoPC(20:3)	2.6	3.4	−0.6	−0.3	0.9	1.1	1	0.2
POS	15.22_547.3573*m/z*	2	HMDB0094688	1-stearoylglycerophosphocholine	3.1	4.4	−0.1	0.1	0.6	1.1	1.5	0.9
POS	15.24_453.2849n	2	HMDB0011503	LysoPE(16:0/0:0)	−1.7	−2	−0.8	−1.2	0	0.6	0.2	0.5
POS	15.27_318.2190n	3	-	Eicosanoid	11.1	11.1	4.6	7.3	0.5	−0.5	0.8	1.3
POS	15.43_521.3476n	2	HMDB0002815	LysoPC(18:1)	0.8	1.2	0	0.2	0	0.1	0.2	0.3
POS	15.43_545.3462n	2	HMDB0010393	LysoPC(20:3)	1.9	2.6	−0.2	0	0.6	0.6	0.7	0.4
POS	15.65_502.3256*m/z*	2	HMDB0010407	LysoPC(P-16:0)	3.9	4.5	0.3	1.4	1.3	1.5	−0.1	0.1
POS	15.66_479.3361n	2	HMDB0010407	LysoPC(P-16:0)	3.1	3.7	−0.1	0.8	0.6	1	0	0.2
POS	15.79_548.3696*m/z*	2	HMDB0010392	LysoPC(20:2)	2.4	3.2	0	0.4	0.2	0.5	0.6	0.3
POS	15.96_547.3629n	2	HMDB0010392	LysoPC(20:2)	2.6	3.5	−0.6	−0.3	1.6	0.2	1.3	1.3
POS	15.99_548.3700*m/z*	2	HMDB0010392	LysoPC(20:2)	1.8	2.6	−0.3	−0.3	0.6	0.2	0.5	0.6
POS	16.80_549.3785n	2	HMDB0010391	LysoPC(20:1)	1.4	2.2	0.1	0.2	0.2	0.2	0.2	0.8
POS	16.90_508.3750*m/z*	2	HMDB0013122	LysoPC(P-18:0)	5.3	6	0.4	1.2	2.4	1.7	0	1.5

**Table 4:** Top 10 metabolic pathways identified after pathway analysis performed on the 205 endogenous deregulated metabolites after HTP exposure using MetaboAnalyst (v5.0). Enrichment method: hypergeometric test; topology analysis: relative-betweenness centrality; pathway library: mammals, Homo sapiens (KEGG).

Pathway	Total Number of Compounds in the Pathway	Hits	*p*-Value	Adjusted *p*-Value (FDR)	Pathway Impact Value Calculated Based on Pathway Topology Analysis
Amino sugar and nucleotide sugar metabolism	37	3	0.009	0.45	0.15
Pyrimidine metabolism	39	3	0.010	0.45	0.07
Purine metabolism	65	3	0.042	0.93	0.13
Glutathione metabolism	28	2	0.044	0.93	0.02
Glycerophospholipid metabolism	36	2	0.070	0.98	0.03
Phosphonate and phosphinate metabolism	6	1	0.071	0.98	0.50
Tryptophan metabolism	41	2	0.087	0.98	0.02
Ascorbate and aldarate metabolism	8	1	0.094	0.98	0.00
Nicotinate and nicotinamide metabolism	15	1	0.169	1.00	0.00
Starch and sucrose metabolism	18	1	0.200	1.00	0.01

## Data Availability

The data presented in this study are available from the corresponding author upon request.

## Abbreviations

ALI	Air–liquid interface
CCS	Collision cross-section
e-cigs	Electronic cigarettes
ESI	Electrospray ionization
FDR	False discovery rate
GSSG	Oxidized glutathione
HMDB	Human metabolome database
HO	Heme oxygenase
HRMS	High-resolution mass spectrometry
HTPs	Heated tobacco products
IMS	Ion mobility spectrometry
LC	Liquid chromatography
LDH	Lactate dehydrogenase
Log2(FC_D1_)	Log2(FC) D0 vs. D1
Log2(FC_D2_)	Log2(FC) D0 vs. D2
LysoPC	Lysophosphatidylcholine
LysoPE	Lysophosphatidylethanolamine
*m*/*z*	Mass-to-charge ratio
Mb-18W	Modbox e-cig model set at 18 W
Mb-30W	Modbox e-cig model set at 30 W
MS	Mass spectrometry
Nrf2	Nuclear factor erythroid 2-related factor 2
PBS	Phosphate buffer solution
PLA2	Phospholipase A2
QC	Quality control
QToF	Quadrupole time-of-flight
ROS	Reactive oxygen species
Rt	Retention time
TCA cycle	Tricarboxylic acid cycle
UPLC	Ultra high-performance liquid chromatography
VIP	Variable importance in the projection
PLS-DA	Partial least-squares discriminant analysis
